# Structure-activity relationship analysis on the basis of matched molecular pairs

**DOI:** 10.1186/1758-2946-6-S1-O14

**Published:** 2014-03-11

**Authors:** Anne Mai Wassermann

**Affiliations:** 1 Novartis Institutes for Biomedical Research, Cambridge, MA, 02139, USA

## 

Matched molecular pairs (MMPs), i.e., pairs of compounds that are related to each other by a specific molecular transformation, have become an integral tool of drug discovery [1,2]. Generally spoken, matched molecular pair analysis (MMPA) aims at the extraction of all MMPs from a set of compounds and their association with calculated or measured property changes. Using public bioactivity data, we have used MMPs as a consistent reference framework to identify sets of chemical replacements that either have the propensity to induce large-magnitude potency changes or tend to retain compound potency across diverse targets [3,4]. Furthermore, we have extended the concept of MMPs to matched molecular series, i.e., analog series with different molecular core structures but corresponding substitution patterns [5,6]. The identification of series with alternative core structures but similar SAR trends is highly relevant for lead optimization where SAR information from one series that has been explored historically is ideally used to guide compound design efforts for a new chemotype [6].
